# The Ball Welding Bar: A New Solution for the Immediate Loading of Screw-Retained, Mandibular Fixed Full Arch Prostheses

**DOI:** 10.1155/2017/2679085

**Published:** 2017-08-01

**Authors:** Danilo Bacchiocchi, Andrea Guida

**Affiliations:** ^1^Private Practice, Castelfidardo, 60022 Ancona, Italy; ^2^Fundacao Universitaria Vida Crista (FUNVIC), 12400-010 Pindamonhangaba, SP, Brazil

## Abstract

**Purpose:**

To present a new intraoral welding technique, which can be used to manufacture screw-retained, mandibular fixed full-arch prostheses.

**Methods:**

Over a 4-year period, all patients with complete mandibular edentulism or irreparably compromised mandibular dentition, who will restore the masticatory function with a fixed mandibular prosthesis, were considered for inclusion in this study. The “Ball Welding Bar” (BWB) technique is characterised by smooth prosthetic cylinders, interconnected by means of titanium bars which are adjustable in terms of distance from ball terminals and are inserted in the rotating rings of the cylinders. All the components are welded and self-posing.

**Results:**

Forty-two patients (18 males; 24 females; mean age 64.2 ± 6.7 years) were enrolled and 210 fixtures were inserted to support 42 mandibular screw-retained, fixed full-arch prostheses. After two years of loading, 2 fixtures were lost, for an implant survival rate of 97.7%. Five implants suffered from peri-implant mucositis and 3 implants for peri-implantitis. Three of the prostheses (3/42) required repair for fracture (7.1%): the prosthetic success was 92.9%.

**Conclusions:**

The BWB technique seems to represent a reliable technique for the fabrication of screw-retained mandibular fixed full-arch prostheses. This study was registered in the ISRCTN register with number ISRCTN71229338.

## 1. Introduction

In 1982, P. L. Mondani and P. M. Mondani published an article in which they fully described the equipment and techniques necessary for intraoral welding, a welding procedure for intraoral implant abutments, developed to obtain an immediate fixed prosthesis without the need for complex and lengthy laboratory procedures [1]. The method was essentially based on the creation of an electric arc between two electrodes under an argon gas flux [1]. Current scientific literature has validated the use of intraoral welding techniques [2–6]. In 2002, Hruska et al. published a study reporting the results of 1301 immediately loaded implants, 436 of which were used to support fixed partial dentures and full arches built over intraorally welded frameworks [2]. In this paper, the authors reported a rather low incidence of implant failures, with three failed implants (0.7%), one due to fracturing and two due to peri-implantitis [2]. The authors showed how, in cases of extensive reconstruction, intraoral welding had the advantage of simplifying prosthetic procedures and in particular those involving highly disparallel abutments [2]. Intraorally welded frameworks acted as mesiostructures and reduced the incidence of fractures of the provisional prosthesis [2]. More than 20 years after the publication of Mondani's article [1], Degidi et al. published a study on the immediate loading of multiple implants using a preformed bar that was welded intraorally to the implant abutments and that supported a temporary, metal-reinforced bridge [3]. All 192 immediately loaded implants survived, and no prosthetic complications occurred at the level of the provisional prosthesis [3]. This structure proved to be capable of withstanding load better than a temporary restoration without reinforcement, as demonstrated by finite element analysis [3]. In a later work by the same authors [4], intraoral welding proved to be a reliable technique for the rehabilitation of completely edentulous mandibles. The process they described involved the delivery and immediate loading of a full arch prosthesis on the day of surgery, using fixtures with butt-joints and conical implant-abutment connections. Once again, in a further prospective clinical work, Degidi et al. showed the rehabilitation of the fully edentulous mandible by inserting 4 implants, splinted to each other through the intraoral welding of a bar to their titanium abutments [5]. The framework thus obtained was used to support an immediately loaded definitive prosthesis [5]. In brief, 22 patients treated with 88 implants were followed for a total period of one year. At the end of the period, only one implant was lost within a month of insertion, for an implant survival rate of 98.9% [5]. No fractures or alterations occurred to the intraorally welded framework, and no fractures of the prosthetic acrylic resin superstructure were recorded [5]. Finally, in 2013, Degidi et al. reported the 6-year follow-up results of the welding technique for the fabrication of immediately loaded maxillary and mandibular fixed full arches [6]. All the patients in this study were rehabilitated on the same day of surgery with a temporary, immediately loaded prosthesis built on a titanium framework obtained by the intraoral welding of a titanium bar to the implant abutments [6]. In total, there were 124 implants placed in the maxilla and 87 implants placed in the mandible; the fixtures were controlled for up to 6 years after loading [6]. Mean peri-implant bone resorption was measured as 1.39 mm (±0.67) and 1.29 mm (±0.71) for the maxilla and mandible, respectively [6]. The most frequent complication was the fracture of the resin superstructure. Overall, the intraoral welding technique proved to be effective and reliable in allowing the fabrication of immediately loaded prostheses in edentulous patients [6]. Some possible variations to the classic technique of intraoral welding have been presented in recent scientific literature [7–10]. In the most commonly used methods, the diameter of the bar (generally made of titanium grade 2) is chosen based on the distance between the implants, the extent of the arch, and the available prosthetic volumes. The bar is then shaped to be adherent to the titanium cylinders placed on the abutments and is then welded to them [2–6, 8, 10]. The purpose of our present work is therefore to present a new variant of the intraoral welding technique, which can be used to manufacture full arch screw-retained rehabilitations of the edentulous mandible, under an immediate loading protocol. This innovative type of prosthetic rehabilitation, which the authors refer to as the “Ball Welding Bar” (BWB) technique, is characterised by smooth prosthetic cylinders, interconnected by means of titanium bars (grade 4) which are adjustable in terms of distance from ball terminals and are inserted in the rotating rings of the cylinders. All the components are welded and self-posing and do not cause arcing or tension. This paper reports on a study tracking the results obtained two years after immediate loading of a full arch, screw-retained mandibular prosthesis (Toronto bridges) that was screwed onto the new intraorally welded Ball Welding Bars.

## 2. Materials and Methods

### 2.1. Patient Sample

In the period between January 2010 and December 2013, all patients who were referred to two different private dental centres (the private dental clinics of Professor Andrea Guida and Dr. Danilo Bacchiocchi) for rehabilitation using oral implants were considered for inclusion in this prospective clinical study. Patients considered for inclusion were those with

1. complete mandibular edentulism, with functional and aesthetic problems related to the presence of a complete, removable conventional denture (i.e., lack of stability of the complete denture, discomfort during function, and aesthetic embarrassment),
2. irreparably compromised mandibular dentition, due to advanced periodontal disease or destructive/massive tooth decay that made the residual dental elements unrestorable,
3. sufficient bone volume (bone height × width) to allow for the placement of implants of at least 8 mm in length and 3.0 mm in diameter,
4. will to restore the masticatory function with a fixed mandibular prosthesis supported by dental implants,
5. the ability to understand and sign an informed consent form for implant treatment.

Patients excluded from the study were those

1. with general medical conditions/systemic diseases that represented an absolute contraindication to surgical and implant treatment, such as severely immunocompromised patients or severely uncompensated diabetics, patients receiving radiotherapy to the head and neck area or chemotherapy, and patients receiving amino-bisphosphonates intravenously and/or orally,
2. with psychiatric disorders,
3. addicted to alcohol or drugs,
4. who needed bone augmentation procedures with autogenous bone or other bone substitutes, to allow for proper implant insertion,
5. who had previously undergone major regenerative bone surgery, preliminary to the placement of dental implants. Inclusion and exclusion criteria for the present study were also summarized in Table 1.

Cigarette smoking was not an exclusion criterion for enrollment in this study; nevertheless, patients that smoke were informed of the fact that cigarette smoking is a risk factor for the success of implant treatments [11]. All patients received detailed information about the planned therapy, the related risks, and possible alternatives. Patients were enrolled only after signing an informed consent form for implant treatment. Finally, the present clinical work was carried out in compliance with the principles set out in the Helsinki Declaration on Human Experimentation of 2000 (revised 2008). The present clinical study was registered in the ISRCTN, a publicly available register for clinical trials recognized by WHO and ICMJE, with number ISRCTN71229338.

### 2.2. The Ball Welding Bar (BWB) Concept

**Figure 1:**
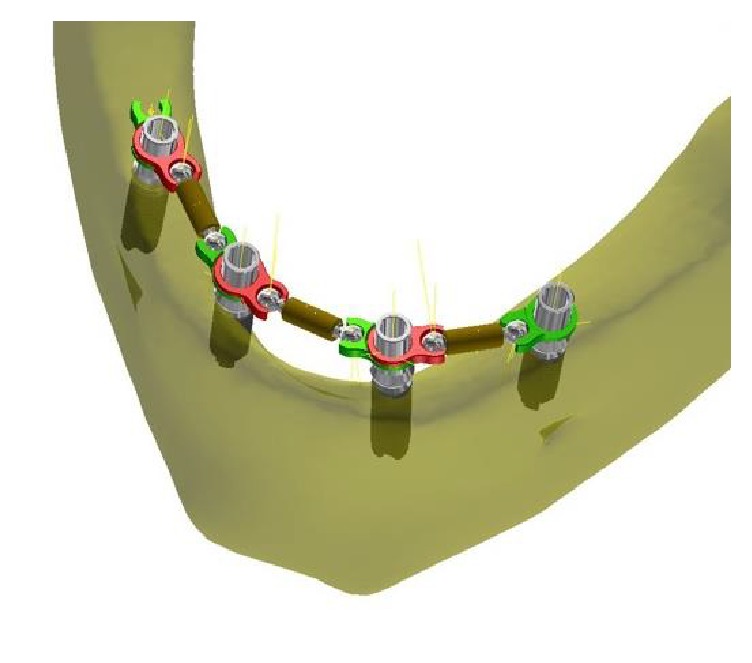
The BWB (Ball Welding Bar) consists of smooth prosthetic cylinders, interconnected by means of titanium bars (grade 4) adjustable in distance with ball terminals, which are inserted in the rotating rings of the cylinders. All the components are welded and self-posing, without arcing nor tensions. This BWB has been patented by the authors (patent number AN2014A000111 and variants).

The BWB (Ball Welding Bar) consists of smooth prosthetic cylinders, interconnected by means of titanium bars (grade 4) which are adjustable in terms of distance from ball terminals and are inserted in the rotating rings of the cylinders. All the components are welded and self-posing and do not cause arcing or tension (Figure 1). This prosthetic device has been patented by the authors (patent number AN2014A000111 and variants).

### 2.3. The Implants Used in This Study

**Figure 2:**
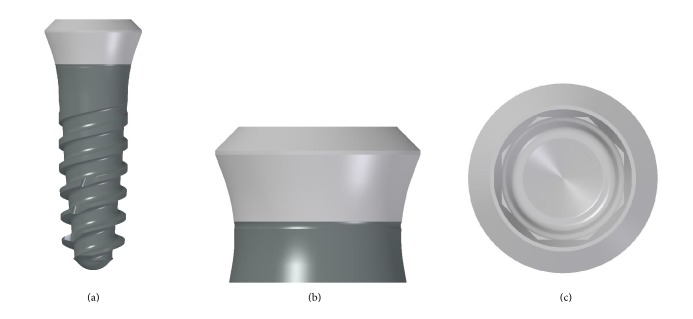
Drawing of the tapered, double lead threads implant used in this study. (a) Apical threads, deeper and cutting, favour insertion, and initial stability, whereas squared coronal thread enhances bone condensation. (b) Back-tapered collar provides excellent cortical bone management, improving soft tissue support. (c) Octagonal conical connection (2 mm in depth with 8° cone) that guarantees an excellent seal, reducing the risk of micromovements between the implant and the abutment.

The fixtures used in the present work (BT Safe Bone Level®; Biotec BTK, Povolaro di Dueville, Vicenza, Italy) were made of titanium grade 4 (ASTM F67dISO 5832-2). These were tapered implants with double lead threads (Figure 2) and a hexagonal conical connection (11°) and integrated platform switching [12]. The dual acid etched (DAE) surface of these implants was the result of treatment with a mixture of strong inorganic acids (H2SO4, H3PO4, HCl, and HF) [13]. The implants were then rinsed and washed with distilled water, to neutralize acid residuals. Finally, implants were taken to a cleaning room (ISO 7 class) to be decontaminated through a plasma spray decontamination process, in an argon atmosphere.

**Figure 3:**
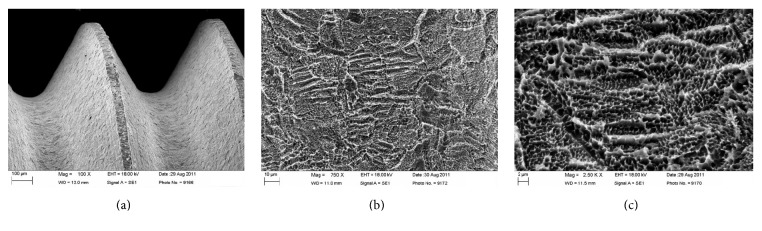
Scanning electron microscopy (SEM) evaluation of the dual acid etched (DAE) implant surface. The surface (a) presented micron-sized shallow cavities uniformly covered by submicroscopic pitting (b) limited by razor-sharp cusps and edges (c).

The DAE implant surface (Figure 3) had the following roughness parameters:

- Ra (arithmetic mean of the absolute height of all points) = 1.12 (60.41) *μ*m,
- Rq (square root of the sum of the squared mean difference of all points) = 1.34 (60.69) *μ*m,
- Rt (difference between the highest and the lowest points) = 3.86 (61.40) *μ*m [13].

### 2.4. Surgical and Prosthetic Phases

**Figure 4:**
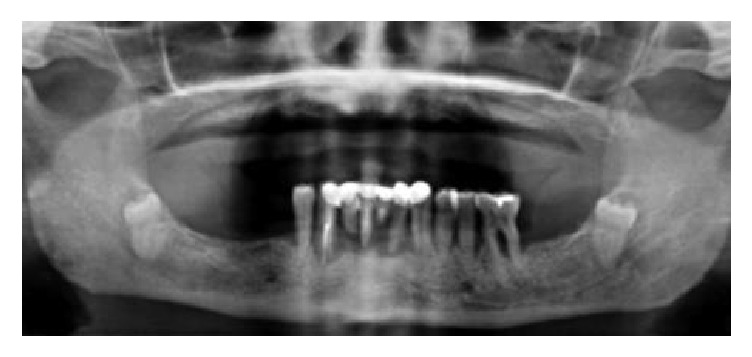
Preoperative situation. A 52-year female patient in good general health had a complete removable denture in the maxilla and a severe acute periodontitis in the mandible, with several teeth with reduced bone support and therefore high mobility. The patient asked for a full arch implant supported rehabilitation of the mandible, possibly involving an immediate loading protocol.

**Figure 5:**
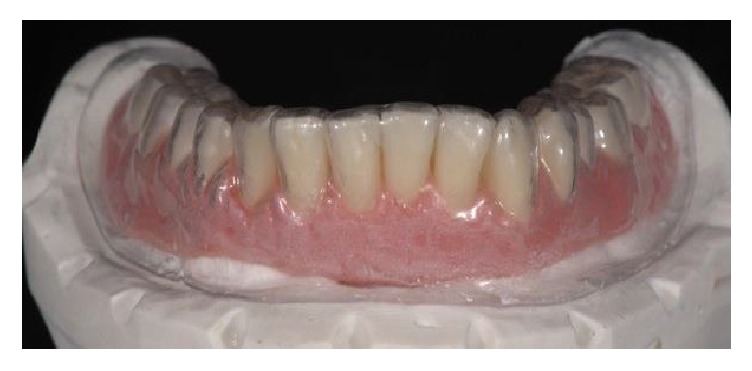
Prior to surgery, a complete lower denture was fabricated in the dental laboratory, made in composite resin with a transparent vacuum-formed template.

**Figure 6:**
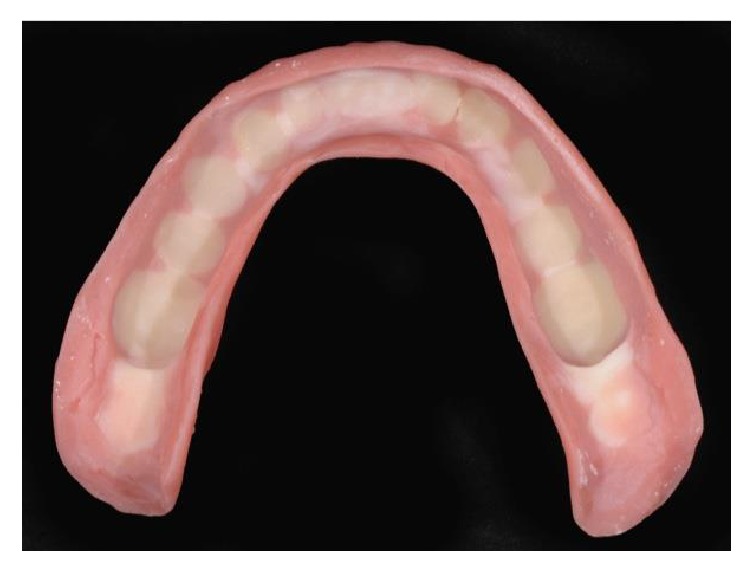
The lower denture was emptied internally, in order to subsequentially accommodate inside the future intraorally welded titanium framework. The flanges and the supports in the retromolar areas were preserved, in order to facilitate the positioning in the postwelding phase.

**Figure 7:**
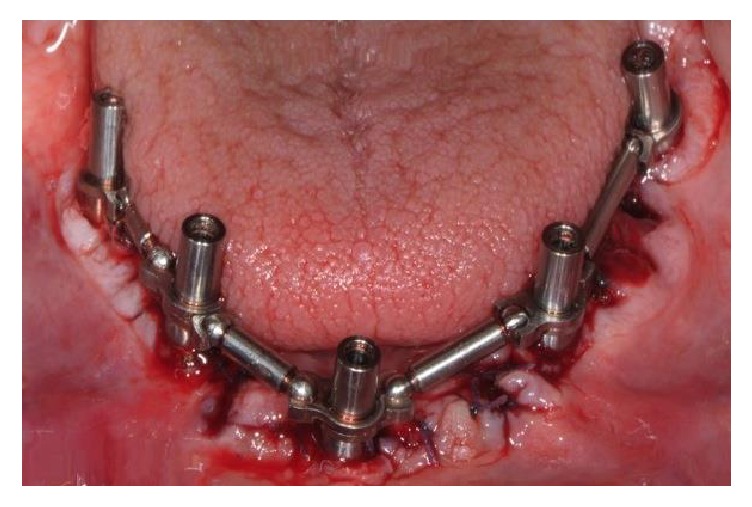
The prosthetic cylinders in titanium were screwed: over these cylinders, the adjustable rings and the rotating spheres were adapted both horizontally and vertically, paying attention to remain well above the bone crest.

**Figure 8:**
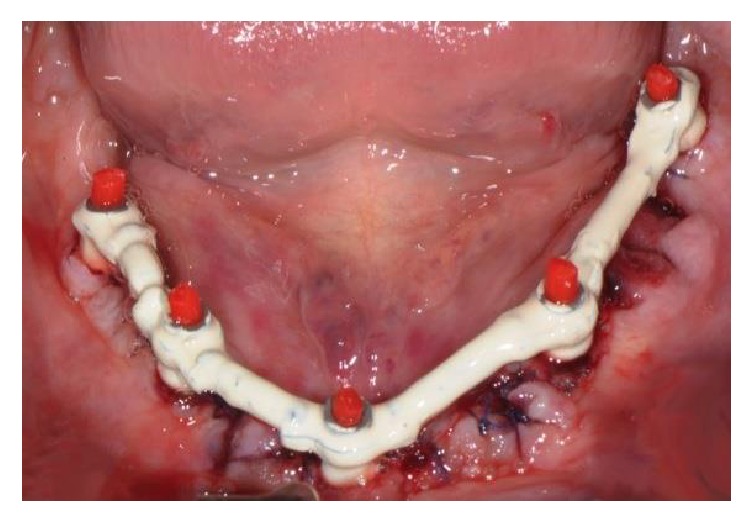
After the intraoral welding, the self-locating bar was removed from the mouth and finished, blasted, and then covered with a white opaque composite resin. The bar was subsequently repositioned onto the MUA abutments.

**Figure 9:**
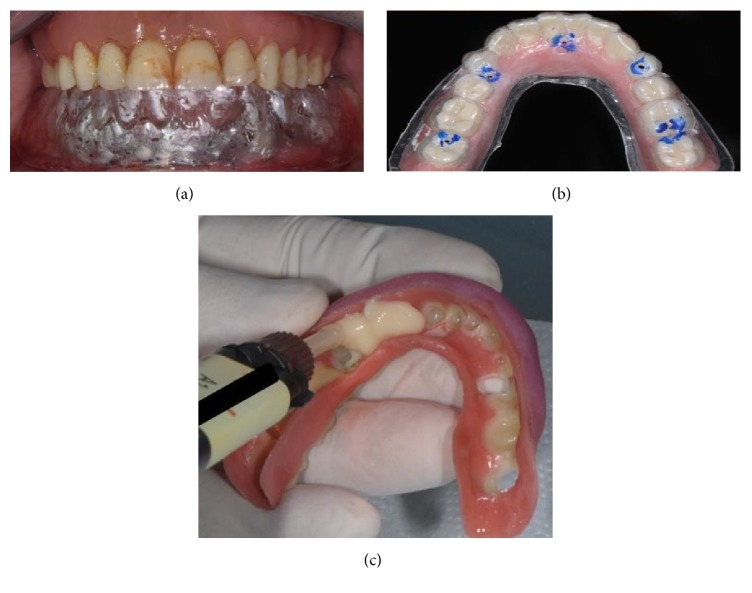
The vacuum-formed template was placed over the bar to control the occlusal fairness and to verify the previously measured vertical dimension (a). Holes were made on the prosthesis, using the vacuum-formed template already perforated in the direction of the cylinders, in order to access to the cylinders themselves (b). The prosthesis was relined with composite resin flow (c).

**Figure 10:**
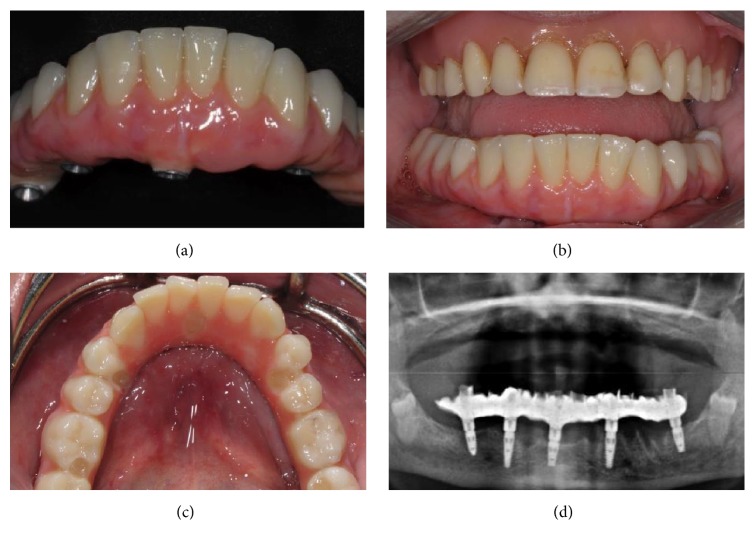
The fabrication of the prosthesis is completed in the laboratory (a), which after a few hours return the Toronto Bridge to the dentist for clinical application. The clinician delivers the prosthesis to the patient (b), after a careful check of the occlusion that must be perfectly balanced; then, the clinician closes the holes of the cylinders with Teflon soaked in chlorhexidine 5% and light curing flowable composite resin (c). The panoramic radiograph shows the titanium framework and the perfect adaptation of this structure on implant abutments (d).

A preliminary clinical and radiographic evaluation with panoramic radiographs (Figure 4) preceded the surgery. Where needed, a cone beam computed tomography (CBCT) scan (I-Max Touch 3D®, Owandy Radiology, Oxford, CT, USA) was performed, in order to collect all anatomical information for optimal surgical and prosthetic planning. The digital imaging and communication in medicine (DICOM) files from CBCT were imported into three-dimensional (3D) reconstruction software, where the surgical and prosthetic planning was performed, and the feasibility of the protocol was investigated. The present protocol involved the fabrication of screw-retained, complete mandibular dentures (Toronto bridge) supported by 5 dental implants. Two implants had to be inserted in the molar areas (3.6 and 4.6, resp.), two in the first premolar areas (3.4 and 4.4, resp.), and the last one in midline or otherwise in the incisal areas (3.1 or 4.1). The prosthesis had to be immediately loaded, supported by multiunit abutments (MUA) splinted together with a Ball Welding Bar (BWB). Prior to surgery, a complete lower denture was fabricated in the dental laboratory, from composite resin with a transparent vacuum-formed template (Figure 5). In order to achieve this, impressions were taken and casts were developed and mounted in an articulator, with bite-in wax for definition of the proper occlusion and the selection of the colour and the shape of the teeth. This lower denture was then hollowed in order to be able to accommodate the intraorally welded titanium framework. Flanges and supports were kept on the retromolar areas to facilitate positioning in the postwelding phase (Figure 6). Surgery commenced after infiltration of local anaesthesia. A full-thickness flap was raised after a crestal incision was performed, and two releasing vertical incisions were made. In the case of partially edentulous patients, the nonrestorable teeth affected by severe periodontal disease or decay were then removed, taking care not to damage the socket walls. This was followed by the preparation of the implant sites and the deepening of the apex of the socket (3-4 mm). In the case of fully edentulous patients with completely healed ridges, the preparation of the implant sites was performed in accordance with manufacturer recommendations, taking into account the clinical situation. The implants were then inserted in both extraction sockets and healed sites (the final insertion torque of the fixtures had to be ideally > 55 N/cm). The prosthetic phase started immediately with the placement of multiunit abutments (MUA) with a transmucosal height of 3 mm over the implants. The aforementioned MUA were screwed gently with a torque of 30 N/cm. In all immediate postextraction implants, the remaining gaps between the implant and the walls of the socket were filled and packed with a resorbable *β*-tricalcium phosphate [14] regenerative material (OXOFIX®, Biotec BTK, Povolaro di Dueville, Vicenza, Italy BTK, Italy). Finally, sutures were made. The prosthetic procedure went as follows. The prosthetic titanium cylinders provided by systematic BWB were first screwed in. Over these cylinders, the adjustable rings and rotating balls were adjusted both horizontally and vertically, taking care to remain well above the crest, and not to occupy extracrestal spaces (Figure 7). The self-locating bar was welded into the mouth, then removed, and finished; after blasting, it was covered with a white opaque light curing composite resin and repositioned onto the MUA abutments (Figure 8). The vacuum-formed template was placed over the bar to control the occlusal fairness and to verify the previously measured vertical dimensions (Figure 9(a)). Holes were made on the prosthesis using the vacuum-formed template which was already perforated in the direction of the cylinders (Figure 9(b)) in order to give access to the cylinders themselves after relining with composite resin flow (Figure 9(c)). Adhesive (Universal Futura B®, Voco, Cuxhaven, Germany) was positioned within the prosthesis and light cured, and the prosthesis was filled with a composite dual fluid (Rebilda, Voco, Cuxhave, Germany) and positioned above the bar. The prosthesis was self-centring, because it had all the support on the retromolar areas and the vestibules like a conventional full denture; therefore it found its natural position and occlusal vertical dimension without any error or unwanted movement (Figure 10(a)). When the dual composite resin was completely polymerized, the prosthesis was removed and sent to the laboratory, which after a few hours finished and returned it. The prosthesis was then delivered to the patient in less than 6 hours (Figures 10(b), 10(c), and 10(d)). The occlusion was carefully checked because it had to be perfectly balanced, and the holes were closed again with Teflon cylinders soaked in chlorhexidine 5% and a flowable light-cured composite. The patients received detailed instructions about oral hygiene and home care procedures and were enrolled in a series of scheduled follow-up controls, every 4 months, for professional oral hygiene sessions and clinical monitoring of their rehabilitation.

### 2.5. Outcome Variables and Statistical Evaluation

The main outcome variables for the present study were implant survival and the prosthetic success. With regard to implant survival, an implant was classified as “surviving” if it functioned regularly at the end of the study, two years after its placement and functional loading. In all cases in which the implant had to be removed, the fixture was defined as “failed.” The causes for implant failure were as follows:

1. mobility for lack of osseointegration, which occurred in the early healing period (not later than 4 months after insertion) but in the absence of symptoms/signs of infection,
2. infection of the peri-implant tissue (peri-implantitis) that caused massive bone loss and subsequent loosening of the implant [15]. The threshold for diagnosis of peri-implantitis was a probing depth ≥4 mm, bleeding on probing, and/or pus secretion associated with evidence of radiographic bone loss (>2.5 mm) [15],
3. progressive severe bone loss (>2.5 mm per year) in the absence of specific symptoms/signs of infection,
4. fracture of the implant body.

With regard to prosthetic success, a prosthesis was considered successful when no adverse events (such as fractures/alterations of the resin superstructure and of the intraorally welded titanium framework) occurred [3–6, 16]. At the end of the study, after 2 years of functional loading, all relevant patient data (gender, age at surgery, and smoking habits), implant information (position, length, and diameter), and prosthesis information were collected in an Excel spreadsheet. Means (±SD) ranges, medians, and confidence intervals (95%) were calculated for quantitative variables and absolute and relative (%) frequency distributions were obtained for all qualitative variables. Implant survival and prosthesis success were finally calculated at the patient level, which meant that if even a single implant out of five failed, the procedure on that patient was classified as a failure, and in the presence of even a single prosthetic complication, the prosthesis could not be considered successful [17].

## 3. Results

In total, 42 patients (18 males and 24 females) were included in the present study. The mean age of patients was 64.2 (±6.7), range 54–79 years, median 63.5 years, and confidence interval (95%) 62.1–66.2. Among these patients, 12 (28.5%) were smokers. Overall, 210 fixtures were inserted to support 42 screw-retained, full arches restorations (Toronto bridges) in the edentulous mandible. The distribution of the implants was as follows: 18 fixtures (8.6%) were 3.7 mm in diameter × 12 mm in length, 40 fixtures (19%) were 3.7 × 14 mm, 46 fixtures (21.9%) were 3.7 × 16 mm, 18 fixtures (8.6%) were 4.1 × 12 mm, 48 fixtures (22.9%) were 4.1 × 14 mm, and 40 fixtures (19%) were 4.1 × 16 mm. The positions of the implants were the following: 84 molars (40%), 84 premolars (40%), and 42 incisors (20%). At the end of the study, only 2 fixtures were lost (2/210: 0.9%) during the first months after placement, in a single patient: the implant survival rate was 97.7% (patient-based). With regard to complications, during the follow-up, 5 implants suffered from peri-implant mucositis and 3 implants suffered for peri-implantitis: these fixtures were, however, successfully treated with dedicated professional oral hygiene sessions and no further biological problems were registered at the end of this work. With regard to prosthetic complication and therefore prosthetic success, 3/42 of the prostheses required repair for fracture (7.1%): this was considered a major complication. In addition, 4 multiunit abutments (1.9%) became loose during the entire follow-up: these were reinserted and screwed and no other abutment loosening was encountered in this study. The loosening of the multiunit abutments was considered as a minor complication.

## 4. Discussion

In recent years, several clinical studies have reported excellent results obtained using intraoral welding techniques for the rehabilitation of completely edentulous mandibles with screw-retained full arch immediately loaded prostheses and Toronto bridges [2–6]. Similar results have also been reported for procedures on the fully edentulous maxilla [6, 18, 19], giving the impression that intraoral welding can be successfully used for the rehabilitation of edentulous patients. In particular, Degidi et al. published a paper [18] in which 30 patients received 3 axial and 4 tilted implants in the edentulous maxilla and were rehabilitated with an immediately loaded definitive prosthesis achieved using intraoral welding. Patients were followed for a period of 3 years during which the 210 implants inserted were checked, and implant failures, marginal bone resorption around the fixtures, and prosthetic problems were carefully registered [18]. At the end of the study, three implants had biological problems, giving a success rate of 97.8% for axial implants and 99.2% for tilted implants, respectively. The mean marginal bone resorption was 0.92 mm (±0.75) for the axial and 1.03 mm (±0.69) for the tilted implants [18]. No fractures or alterations of the intraorally welded titanium framework occurred, for a prosthetic success of 100% [18]. One of the obvious advantages of the intraoral welding technique is the ability to rehabilitate in a very short timeframe and with limited costs fully edentulous patients, without going through lengthy and complex laboratory phases [2–6, 18]. Recently, some potential alternatives to the traditional technique originally proposed by P. L. Mondani and P. M. Mondani [1] and subsequently recovered by Degidi et al. [3, 5, 6, 18] have been proposed [7, 8]. Albiero and Benato [8] published a case report in which the technique of intraoral welding has been combined with modern guided surgical techniques. Using this “guided-welded approach,” the authors were able to obtain a very precise passive fit of a maxillary complete denture supported by 4 implants, loaded immediately [8]. The passive fit contributed to the optimal healing of the implants and the use of guided surgery allowed for the reduction of surgery time and the adaptation of the bar to the implant abutments [8]. Fornaini et al. [7, 9, 10] have presented another possible variant of the intraoral welding technique, using a laser for the welding of a bar (previously prepared from a technician) to 4 implants placed in an edentulous maxilla. In particular, in their clinical report [7] preceded by an in vitro evaluation, the authors proved that the use of lasers can produce good results in terms of intraoral welding. The theoretical advantages of the use of lasers for the welding are different: lasers is effective on all metals and can be used without filler metal and shielding gas and thanks to the fact that the beam has extremely small dimensions and is well focused (0.6 mm), there is no adverse effect (overheating) on the surrounding tissue [7, 9, 10]. In addition, lasers can be used on all patients (even on patients with pacemakers) [7, 9, 10]. In our present clinical research, we have introduced a further possible variant of the classical intraoral welding technique procedure: the so-called “Ball Welding Bar” (BWB) technique. The BWB technique represents a new, simple treatment option for the fabrication of a screw-retained Toronto Bridge that has predictable results. The mechanical properties of this new bar (made of titanium grade 4) and the original assemblage designed and patented by the authors allow for the rapid fabrication of prostheses with no tension. This means that there will be good adaptation when using a variety of loading protocols (including immediate functional loading). Overall, 210 fixtures were inserted to support 42 screw-retained, full arches restorations (Toronto bridges) in the edentulous mandible. At the end of the study, only 2 fixtures were lost (2/210: 0.9%) during the first months after placement, in a single patient: the implant survival rate was 97.7% (patient-based). With regard to prosthetic complication and therefore prosthetic success, 3/42 of the prostheses required repair for fracture (7.1%): this was considered a major complication. The prosthetic success was therefore 92.9%. The main advantages of our method are that it makes it easy to centre the bars over the bone crest (for ease of orientation of the components in the vertical and horizontal dimensions), allows for fine regulation, and makes it easy to solder the framework without distortions. Finally, the procedure is very rapid and can be managed by a single operator, both of which allow for reduced rehabilitation costs. In the present study, we have used tapered implants with a hexagonal conical implant-abutment connection, because these fixtures had all the features necessary to meet the biological and mechanical requirements for immediate loading, both in fully healed edentulous ridges and in postextraction sockets [12]. The tapered design with double lead threads, in fact, allows the surgeon to obtain an excellent primary implant stability even in difficult clinical conditions [12], such as in the case of postextraction sockets [20]. At the same time, the dual acid etched surface of these implants has the potential to accelerate bone healing, as demonstrated by a recent histologic/histomorphometric human study [13]. In modern oral implantology, the presence of an adequate macrostructure (thread design) and microstructure (surface roughness) is considered critical for functional immediate loading [21–23]. Finally, new materials (such as composite resins) are now available for the fabrication of fixed full arch mandibular dentures [24, 25]. These composite resins can effectively replace more conventionally used materials (i.e., acrylic resins) because they offer greater hardness compared to conventional acrylic resins. In addition, they are better in terms of aesthetics, as they come in different shades of pink to mimic the colours of the gum [24, 25]. These features allow the technician to fabricate a prosthesis composed of a single material, in which the titanium framework/bar is incorporated and welded to the cylinders and the abutments. The resin composite, besides being stronger and more aesthetically pleasing than acrylic resin, has the potential to preserve the occlusion stability over time, and the maintenance and repair are similar to those of a common dental composite [25]. In our present study we have used these materials, and we have obtained excellent functional and aesthetic outcomes. Our present study has limitations, for example, the limited number of patients treated and prostheses fabricated; therefore further studies on a larger sample of patients are needed to confirm the positive clinical outcomes reported here. In addition, the present study has tracked outcomes for only two years and it is necessary to follow the progress of these patients in the long-term, before more specific conclusions can be drawn about the reliability of the new and innovative BWB technique.

## 5. Conclusions

Fulfilling patient demands for an immediate functional recovery is the main goal of modern dentistry. The novel “Ball Welding Bar” technique proposed in the present study is simple and makes it possible to immediately load a definitive screw-retained fixed full arch prosthesis, without the use of a provisional prosthesis. In our present study, 210 fixtures were inserted to support 42 mandibular screw-retained, fixed full arches restorations (Toronto bridges). After two years of loading, 2 fixtures were lost (2/210: 0.9%) during the first months after placement, in a single patient: the implant survival rate was 97.7% (patient-based). With regard to prosthetic complication and therefore prosthetic success, 3/42 of the prostheses required repair for fracture (7.1%): this was considered a major complication. The prosthetic success was therefore 92.9%. Further studies are needed to confirm these positive outcomes.

## Figures and Tables

**Table 1:** Inclusion and exclusion criteria for enrollment of patients in the study.

Inclusion criteria	Exclusion criteria
(1) Complete mandibular edentulism, with functional and aesthetic problems related to the presence of a complete, removable conventional denture.	(1) Severely immunocompromised status, severely uncompensated diabetes, radiotherapy of head and neck area, chemotherapy, and treatment with intravenous and/or intraoral amino-bisphosphonates.

(2) Irreparably compromised mandibular dentition, due to advanced periodontal disease or destructive/massive tooth decay that made the residual dental elements unrestorable.	(2) Psychiatric disorders.

(3) Sufficient bone volume to allow for the placement of implants of at least 8 mm in length and 3.0 mm in diameter.	(3) Alcohol and/or drugs addition.

(4) Will to restore the masticatory function with a fixed mandibular prosthesis supported by dental implants.	(4) Need for bone augmentation procedures with autogenous bone or other bone substitutes, to allow for proper implant insertion.

(5) Ability to understand and sign an informed consent form for implant treatment.	(5) Previous interventions of regenerative bone surgery.
